# Cocaine-Injections for the Production of Anæsthesia

**Published:** 1893-10

**Authors:** Anthony Bleichsteiner

**Affiliations:** Gratz, Austria


### COCAINE-INJECTIONS FOR THE PRODUCTION OF ANÆSTHESIA.[1]

BY DR. ANTHONY BLEICHSTEINER, GRATZ, AUSTRIA.

UNDER this same title I presented in the year 1889 in Paris, at the "Congrès Dentaire International," the results of my experience with cocaine-injections for the purpose of painless extraction of teeth. Up to that time I had more than three thousand injections with five-per-cent. solutions of cocaine hydrochlorate. To-day I shall speak about the experience I have collected in executing more than fourteen thousand injections. I shall give you a description of,—

1. The preparation I use.
2. The character of the solution.
3. What quantities of cocaine hydrochlorate have been used and for how many injections.
4. My syringe.
5. My method of injecting.
6. My opinions concerning the intoxications following cocaine injection.

In December, 1886, I made the first trials with cocaine hydro-

---

[1] Read before the World's Columbian Dental Congress, Chicago, August, 1893.

chlorate (Merck). I began with twenty-per-cent. solutions. After several trials I diminished them to ten-per-cent. solutions. After 1887 I used five-per cent., but since March, 1892, only three-per-cent. solutions.

At first I renewed the solution immediately before every injection, but when I came to execute every extraction with cocaine anæthesia, I prepared the cocaine solution in quantities of ten grammes fluid each, and I used for the sterilization of the solutions corrosive sublimate. I took ten grammes of sublimate solution, containing one gramme of sublimate to five thousand grammes of distilled water, and dissolved in it five decigrammes as long as I used five-per-cent. solutions; now I dissolve in ten grammes of the same sublimate solution three decigrammes of cocaine hydrochlorate, by which I obtain a three-per-cent. solution of cocaine hydrochlorate. A drop of this solution contains three milligrammes of cocaine hydrochlorate.

In the manner previously mentioned, since the year 1887 I have used six hundred grammes of cocaine hydrochlorate (Merck) exclusively for injections, and according to calculation I have made fourteen thousand injections with it.

Four hundred and fifty grammes were used for five-per-cent. solutions until the end of March, 1892. From that time up to the present day one hundred and fifty grammes of three-per-cent. solution have been used, which make five thousand grammes injection-fluid of three per cent. Nine thousand and five thousand are fourteen thousand grammes of cocaine solutions (or fourteen litres), with which, by exact calculation from the year 1887 up to this date, sixteen thousand injections with cocaine have been applied.

I used the "Pravaz" syringe only for a short time for the injections. My syringe as now constructed consists of a glass cylinder encased in hard rubber; its pipe is S-shaped, and in such a way that the prolongation of the end of the pipe forms with the prolongation of the axis of the glass cylinder an acute angle of twenty to thirty-five degrees. The piston-rod of the syringe is divided into ten parts, so that one stroke discharges one decigramme of fluid or a drop. The injection-needles have different lengths, ten and twenty millimetres respectively, in order to be able to adapt them more easily to the different dimensions of the upper and lower jaws. Their sharpened ends are as short as possible, about two millimetres; therefore the length of the puncture can be very short, which is most advantageous. On the handle of the syringe there are two projections, for the forefinger and middle finger.

I will now mention my method of quickly preparing the solution of ten grammes of injection-fluid. I have vials with a wide neck, which will contain ten grammes of fluid, exactly. The vial is filled with the sublimate solution already mentioned, 1 to 5000. I now add three decigrammes of cocaine hydrochlorate, which is weighed in paper cases. By shaking the solution it is made ready for use in a few seconds. The filling of the syringe is done by dipping it without the injection-needle into the solution, and then pulling the piston-rod.

I inject only into the gums near the tooth to be extracted, and I always try to maintain the injection-needle parallel with the curve of the jaw. This is very important, for if I direct the point too much against the epithelial side the uppermost layer of the epithelium is lifted up by the slightest pressure on the piston-rod, in the form of a transparent blister. Such injections are of no value at all. But if I put the needle too much towards the jaw, then I discover that I have struck the socket. I may press as long as I like and not get anything out of the syringe. The piston-rod remains immovable, and in trying to overcome the obstacle by forced exertions either the piston-rod or the glass cylinder breaks, as I have sometimes experienced. I must therefore hold the syringe after the manner of a writing-pen, and force it between the periosteum and the socket. At this place the injection-fluid is pressed by the tension of the periosteum into the bone-tissues of the alveolar wall, and from there into the periosteum. The injection proves to be successful by the gums growing more and more pale, and the formation of a transparent circumscribed blister, which resembles very much a ranula swelling.

Four horizontal and four vertical punctures, of one drop each, generally suffice. At the mesial labial papilla of the gums I also inject two or three millimetres distant from the gums, pricking towards the distal-labial edge. If the injection has succeeded well after the first horizontal puncture, of which the growing paleness of the gums is a sure criterion, the next puncture being made in the pale place, it is not felt by the patient. In this second puncture the needle is again moved in the direction of the distal-labial edge of the gums, and after another injection has been successfully made, the whole labial surface of the gum will have grown pale, perhaps including even the two edges of the gums, mesial and distal from the tooth. In the same way I prick the lingual edge of the alveolar wall, first horizontally from the mesial-lingual edge of the gums, and then again within the reach of the pale spot resulting from the first injection. The punctures and their effects must be continually controlled by means of the mouth-mirror, and the growing paleness of the gums must be carefully observed. If during the injection considerable of the injection-fluid should flow back along the injection-needle, then the injection must be immediately interrupted; a new puncture and new injection must be made a little distant from the first and in another direction. In order to avoid swallowing the cocaine solution, which may have flowed back, and the anæsthesia and paresis of the uvula connected with it, which usually causes a tendency to vomit, I make the patient rinse his mouth immediately after every puncture, and only then do I continue the process.

If, by the horizontal punctures, the gums grow pale all around, then I proceed to make the vertical or the parallel punctures longitudinal to the root or roots of the tooth. I generally make four of them, successively; first the labial-mesial, the labial-distal, then the lingual-mesial, and the lingual-distal puncture. Especially with the lingual-vertical punctures the assistance of the mouth-mirror is indispensable. At every puncture parallel to the length of the tooth the patient is immediately asked whether he feels the puncture, and only in the affirmative case shall the injection be effected. If in case all four vertical punctures have been still felt, the injection must be every time vertical, then it is desirable to make a few more vertical trial-punctures in the immediate space between the places where the punctures have been made, in order to be quite sure that all around the socket of the tooth to be extracted there is no sensitive place to be found for the needle. If a difficult extraction is foreseen, I inject also the septum of the gums between the adjacent teeth in the form of vertical punctures. I have now thoroughly distributed the injection-fluid all around the socket. The length of the horizontal punctures should not exceed five millimetres, while the vertical punctures shall be made as long as possible, at least above ten millimetres. Many authors advise to wait from five to ten minutes after the injection has been made before extracting the tooth. I consider this superfluous for the anæsthesia, and I proceed immediately after the injection, and to this I ascribe the circumstance that I have had to experience comparatively very few and very slight accidents.

Intoxications after cocaine injections only occur in my experience if of twenty- or ten-per-cent. solutions half or even a whole syringeful has been injected. In this case, if the solution be twenty per cent., either one decigramme or two decigrammes of cocaine hydrochlorate have been injected; but with ten-per-cent. solutions, five centigrammes or one decigramme of cocaine. These are doses which are too high under all circumstances. I only had instances of real intoxication in the beginning of my cocaine injections, and as long as I injected such doses. I then found the following symptoms: Dilated pupils, dryness of the throat and mucous membrane, tendency to vomit, a feeling of giddiness, tingling in the ears, a slow pulse, which could easily become pressed and stopped, cold perspiration on the forehead and face, on the hands or over the whole body, a high degree of anæmia of the face, etc. In particularly alarming cases, interruption of the sensory function, lethargy, decay of the bodily strength, and collapse.

I have in the beginning often mistaken for intoxication hysterical paroxysms or even apoplectic fits. My further experience has taught me to distinguish exactly the intoxications from these nervous fits. When, after proper treatment, the alarming symptoms had been overcome, I asked the patient, or those who had accompanied him to my house, whether these had made their appearance at certain intervals or occasions, and my question was always answered affirmatively.

I have found few reasons to believe in these convulsions as the consequence of the procedure, and have almost always been able to range the respective description into the series of one of the reflex symptoms already mentioned. Since I have used three-per-cent. solutions, the accidents described answered more the species of reflex actions. I have rarely injected fifty milligrammes,—that is, the whole contents of a syringe,—and it may be that at one or another time too little was injected, and so with very nervous persons these nervous spasms may have been caused by a feeling of pain from the extraction. That this with nervous persons can be the cause of the reflex symptoms has often been proved. I remember a case when three sound and firmly-fixed teeth were extracted for a very nervous elderly lady at one sitting. After an injection of two and a half syringefuls of a five-per-cent. solution, one hundred and twenty-five milligrammes of cocaine hydrochlorate, not the slightest bad consequences were to be noticed. Two days later I wished to remove a very loose root of a central incisor for this same lady, and I injected only two drops of a five-per-cent. solution,—that is to say, ten milligrammes. After extracting she fell back in the chair and her face lost its color, followed by convulsions of the muscles of the face, the arms, the legs, and of the trunk. The color of the face changed alternately from paleness to redness; clonic spasms passed gradually over to tonic spasms, by which the muscles of the bottom of the cavity of the mouth were especially violently seized. After this kind of spasms had set in, a secretion of perspiration was to be observed. The sensory function was annulled for a short time; the pulse grew slight and could easily be compressed; the respiration became short but frequent. By a horizontal position, cold poultices applied on the forehead as long as the clonic spasms lasted, loosening of the dress, and doses of cognac, as well as making her swallow ice-cream, she was finally able, after an hour, to sit up a little. The first words she uttered were, "Why, doctor, what pain you have made me suffer. I cannot bear any physical pain at all! Only injuring my finger with a needle, I am sure to be attacked by spasmodic fits."

Taking in consideration that two days before one hundred and twenty-five milligrammes of cocaine hydrochlorate had been applied with the best result imaginable, and that ten milligrammes of cocaine hydrochlorate produced, two days afterwards, such a bad effect, the conclusion is easily justified that the symptoms of spasms were not caused by the doses of cocaine hydrochlorate; the more so as the lady herself informed me of her being subject to hysterical paroxysms. Before anything was known of nitrous oxide and cocaine I noted numerous syncopes, hysterical fits, and similar nervous reflexes. Now, when using only three-per-cent. cocaine solutions, I have but rarely perceived reflex symptoms, never real intoxications.

One must be careful that with very timid and nervous patients the first puncture does not excite them, which can be avoided by brushing the gums with cocaine before the injection is applied and that the first puncture is made very gradually, though as quickly as possible; and after very little of the fluid, not even a whole drop, has been injected, the needle is pushed on a little farther, upon which a greater quantity can be injected at once. With persons of nervous disposition, the dentist must try to quiet their excitement by showing himself perfectly composed and at ease in order to gain their entire confidence and trust. Having thus succeeded, the whole procedure may be properly executed. With a three-per-cent. solution one can only experience nervous reflexes, which, however alarming they may seem, have nothing dangerous in themselves.

